# Synthesis and characterization of a magnetic adsorbent from negatively-valued iron mud for methylene blue adsorption

**DOI:** 10.1371/journal.pone.0191229

**Published:** 2018-02-02

**Authors:** Jiancong Liu, Yang Yu, Suiyi Zhu, Jiakuan Yang, Jian Song, Wei Fan, Hongbin Yu, Dejun Bian, Mingxin Huo

**Affiliations:** 1 Science and Technology Innovation Center for Municipal Wastewater Treatment and Water Quality Protection, Northeast Normal University, Changchun, China; 2 Key Laboratory of Songliao Aquatic Environment (Ministry of Education), Jilin Jianzu Univerisity, Changchun, China; 3 Engineering Lab for Water Pollution Control and Resources Recovery, Northeast Normal University, Changchun, China; 4 School of Environmental Science & Engineering, Huazhong University of Science and Technology, Wuhan, China; Institute of Materials Science, GERMANY

## Abstract

With increasing awareness of reduction of energy and CO_2_ footprint, more waste is considered recyclable for generating value-added products. Here we reported the negatively-valued iron mud, a waste from groundwater treatment plant, was successfully converted into magnetic adsorbent. Comparing with the conventional calcination method under the high temperature and pressure, the synthesis of the magnetic particles (MPs) by Fe^2+^/Fe^3+^ coprecipitation was conducted at environment-friendly condition using ascorbic acid (H_2_A) as reduction reagent and nitric acid (or acid wastewater) as leaching solution. The MPs with major component of Fe_3_O_4_ were synthesized at the molar ratio (called ratio subsequently) of H_2_A to Fe^3+^ of iron mud ≥ 0.1; while amorphous ferrihydrite phase was formed at the ratio ≤ 0.05, which were confirmed by vibrating sample magnetometer (VSM), X-ray diffraction (XRD) and X-ray photoelectron spectroscopy (XPS). With the ratio increased, the crystalline size and the crystallization degree of MPs increased, and thus the Brunauer-Emmett-Teller (BET) surface and the cation-exchange capacity (CEC) decreased. MPs-3 prepared with H_2_A to Fe^3+^ ratio of 0.1 demonstrated the highest methylene blue (MB) adsorption of 87.3 mg/g and good magnetic response. The adsorption of MB onto MPs agreed well with the non-linear Langmuir isotherm model and the pseudo-second-order model. Pilot-scale experiment showed that 99% of MB was removed by adding 10 g/L of MPs-3. After five adsorption-desorption cycles, MPs-3 still showed 62% removal efficiency for MB adsorption. When nitric acid was replaced by acid wastewater from a propylene plant, the synthesized MPs-3w showed 3.7 emu/g of saturation magnetization (Ms) and 56.7 mg/g of MB adsorption capacity, 2.8 times of the widely used commercial adsorbent of granular active carbon (GAC). The major mechanism of MPs adsorption for MB was electrostatic attraction and cation exchange. This study synthesized a magnetic adsorbent from the negatively-valued iron mud waste by using an environment-friendly coprecipitation method, which had a potential for treatment of dye wastewater.

## Introduction

With the trend of urbanization, more and more waste is generated in big cities and must be treated in a renewable and sustainable way. Iron mud waste was generated from the groundwater treatment plant and usually contains 3% solids, in which iron and other impurities, such as aluminum, calcium and waste fibers, were present. Iron mud waste are usually treated by naturally drying at the dumping site [1, 2] and the run-off of toxic compounds from it may contaminate nearby soil and water, a great threat of the environment [3]. With the tightening environmental regulations, chemical coagulation followed by mechanical filtration becomes a dominant method in iron mud disposal before sending to landfill, but the cost is high.

Over the past years, studies have been conducted to recover valuable materials from iron mud at aluminum refineries [4], plating factories [5] and steel company [6], such as pigments for mortar and concrete [5, 7], and adsorbents for removal of heavy metals [4, 8] and dyes [2]. In conversion of iron oxide in iron mud to magnetite for synthesis of adsorbent, hydrothermal treatment is usually used at 260°C with adding iron powder [9], roasting with pyrite [10] or charcoal, or injecting with reducing gas, such as H_2_ [8], methane [11] or natural gas [12]. In our previously studies, a reduced temperature of 180°C was used to convert iron mud to MPs by using glycol as a reduction reagent [2]. However, in all the cases, significant energy is demanded in keeping the high temperatures and long heating hours in synthesis of the magnetic adsorbents. Thus, it is important to find an environment-friendly method to transform iron mud to magnetite. Coprecipitation of Fe^2+^/Fe^3+^ mixed salt under alkaline conditions is cost-effective and scaled up easily, which has been widely used in preparing MPs recently [13]. Akin et al. reported [4] that Fe_3_O_4_ nano-particles were coprecipitated by using FeCl_2_.4H_2_O and Fe^3+^ solution after microwave digestion of iron mud. The resulting adsorbent had a high capacity of 0.4 mg/g in adsorbing arsenate from groundwater. Wu et al. [14] also reported that Fe_3_O_4_ nano-particles were obtained by adding FeSO_4_.7H_2_O to HCl-digested iron ore tailings. Despite the Fe_3_O_4_ particles were successfully synthesized in both studies, the production cost may be significantly increased by adding pure ferrous ions in coprecipitation.

It was reported that ascorbic acid (H_2_A) was applied in Fenton reaction to reduce Fe^3+^, Cu^2+^ and Mn^2+^ ions [15] to produce hydroxide radical for oxidation of organic pollutants [16]. Fe^3+^ in iron mud can be partially reduced by H_2_A to generate Fe^2+^ in MP *in situ* production. Therefore, Fe^3+^ from iron mud waste was the sole iron source without adding pure Fe^2+^ ions. Recently, H_2_A was successfully used by Gupta et al in Fe^3+^/Fe^2+^ coprecipitation [17]. It was also used by Nene et al [18] to reduce Fe(ACAC)_3_ in diphenyl-ether solution but the reactions had to be conducted by refluxing the reactants at a high temperature of 190°C for 1 hour. In the present study, Fe^3+^ was partially reduced by H_2_A after iron mud was dissolved with nitric acid and the magnetic adsorbent was synthesized via Fe^2+^/Fe^3+^ coprecipitation at room temperature. To further reduce the production cost, acid wastewater from a propylene plant was used to replace nitric acid in digestion of iron mud. The obtained MPs were further investigated to remove MB from a synthetic wastewater. To our knowledge, it was for the first time to report to synthesize Fe_3_O_4_ particles at the environment-friendly room temperature by H_2_A reduction of Fe^3+^ ions in low solid content of iron mud for Fe^2+^/Fe^3+^ coprecipitation.

## Materials and methods

We had received approval from the Yatai-Longtan Cement Co. Ltd to collect iron mud form its groundwater plant. This study did not involve human participants, specimens or tissue samples, or vertebrate animals, embryos or tissues. In this study, no specific permissions were required for these locations/activities, and provide details on why this is the case. The field studies did not involve endangered or protected species.

### Materials

Iron mud was acquired from the groundwater treatment plant of Jilin Yatai cement company (China), and vacuum-dried at 80°C for 2h before using for Fe_3_O_4_ synthesis. The composition of iron mud was determined by X-ray fluorescence (XRF, ZSX Primus II, Rigaku, Japan). The major components of iron mud solids were quartz and albite and the content of total iron (Fe^2+^ and Fe^3+^) was 16.6 wt%. Acid wastewater was acquired from the propylene plant of Jilin petrochemical company (S1 Table). Nitric acid and H_2_A were purchased from Sinopharm Chemical Reagent Co., Ltd. (Beijing, China). The chemically pure grade GAC was supplied by Tianjin Fuchen Chemical Reagent Factory (Tianjing, China).

### MPs preparation

0.8 g of the dried iron mud was mixed with 30 mL of 2% nitric acid under magnetic stirring over night. After iron mud was dissolved, the suspension was settled for 5 min before the reddish-brown supernatant was poured into a 50 mL Erlenmeyer flask. The flask was then placed into an anaerobic chamber and H_2_A was added to the supernatant to reduce Fe^3+^ ions. After stirring for 15 min, the pH of the supernatant was adjusted to 9.5 by adding 5% NaOH dropwise. When the supernatant became turbid, the flask was heated at 80 ^o^C for 2 h in a water bath for completing precipitation of MPs. The obtained MPs were washed three times in deionized water. Each wash was carried out for 3 min under 40k Hz ultrasound and the supernatant was removed by centrifuging at 5500 rpm for 5 min after washing. The prepared MPs were vacuum-dried at 40 ^o^C overnight before storing at room temperature.

The effect of H_2_A on the adsorption capacity of the synthesized MPs to MB was investigated by changing the ratio of H_2_A to Fe^3+^ ions (Fe^3+^ ion in iron mud, the same in the following) from 0.01, 0.05, 0.1, 0.15 to 0.2, and the obtained particles were denoted as MPs-1, MPs-2, MPs-3, MPs-4 and MPs-5 respectively. MPs-3 was selected for the following adsorption experiments because of its high adsorption capacity. To reduce production cost, nitric acid was replaced by acid wastewater as the dissolving reagent in MP synthesis, and thus synthesized MPs were named as MPs-3w.

### MB adsorption

#### Sorption kinetics studies

The kinetics studies were conducted by mixing 0.015 g of MPs with 20 mL of 60 mg/L MB in a series of nine groups of 50 mL Erlenmeyer flasks. The flasks were sealed with parafilm and shaken for 2h at 150 rpm at 25 ^o^C in a shaking incubator (HZQ-X300, Yiheng, Shanghai, China). During adsorption, one group of the flasks was taken out from the incubator at a given interval, and MB residue in the supernatant was determined by using a UV-vis spectrophotometer (Purkinje General, China) at 655 nm [19] in the reading range of 0.01 to 0.14, which measured MB concentrations between 0.1 mg/L and 0.8 mg/L.

The adsorption capacity of MPs to MB (*q*_*e*_, mg/g) was calculated using the following equation:

$$q_{e}=\frac{(C_{0}‑C_{e})\times V}{m}$$ (1)

Herein, *C*_*0*_ and *C*_*e*_ were the initial and equilibrium MB concentrations (mg/L); *V* was the volume (L) of the MB solution and *m* was the mass of MPs (g) used in MB adsorption.

#### Sorption isotherm models analysis

The sorption isotherm studies were conducted according to the method described by Ramasamy et al. [19]. The initial MB concentrations were 10, 30, 60, 100, 150, 200 and 300 mg/L, respectively, and the equilibrium time was 2h. All data was the average of triplicate experiments.

#### Regeneration studies

MPs-3 had the highest adsorption capacity among the synthesized MPs and was used for regeneration experiments. After equilibrium adsorption of MB with initial concentration of 60 mg/L, the MB-adsorbed MPs-3 was collected with a magnet and then resuspended in 20 mL of deionized water with pH from 1 to 11 to select the best pH for regeneration, followed by shaking at 150 rpm in a shaking incubator (HZQ-300, Yiheng, Shanghai, China) for 24 hours until the MB concentration stabilized according to Yan et al. [20]. The final MB concentrations in solution were measured based on Ramasamy et al. [19]. The desorption efficiency (*R*_*d*_) was calculated using Eq (2).

$$R_{d}=\frac{C_{d}\times V_{d}}{q_{e}\times m}\times 100\%$$ (2)

Where *C*_*d*_ was the final MB concentration after desorption (mg/L); *V*_*d*_ was the volume of aqueous solution in the desorption (L); m was the mass of MPs-3(g) used in MB adsorption.

After desorption, MPs-3 was magnetically isolated from the solution and washed several times with deionized water until pH = 7, followed by suspending in 60 mg/L of MB for the second round of adsorption. The adsorption-desorption cycles were carried out five times, and the re-adsorption efficiency (*R*_*a*_) was calculated with the following equation.

$$R_{a}=\frac{C_{a}\times V_{a}}{q_{e}\times m}\times 100\%$$ (3)

Where *C*_*a*_ was the equilibrium concentration of MB in solution (mg/L); *V*_*a*_ was the volume of aqueous solution (L).

#### The column study

Column experiment was conducted in a plexiglass column with the inner diameter of 22 mm and the height of 110 mm. The concentration of MB solutions was 100 mg/L. Temperature and the initial pH were 25 ^o^C and 7.2, separately. The flow rate was controlled at 2 mL/min with a peristaltic pump (HL-1D, Huxi, Shanghai, China). At a given interval, the effluent was sampled for MB determination.

#### The pilot study

The pilot experiment was performed by using 2L of synthesized wastewater containing 100 mg/L MB with MPs-3 at concentrations of 0.75, 1.5, 2.5, 5, 7.5 and 10 g/L, respectively, in 5L buckets, on which a shafted propeller (OS20, Dalong, Beijing, China) was mounted and agitated at 300 rpm. After 2 hour, MPs-3 was separated from the supernatant with a magnet and the supernatant was analyzed for MB residue.

### Characterization of MPs

The saturation magnetization (Ms) of MPs and iron mud was determined by a magnetometer (Quantum Design, USA) with a SQUID-VSM system. The XRD patterns were conducted for phase identification using a diffractometer (RAPID-S, Rigaku, Japan) with Cu Kα radiation. The crystallization degree of MPs was analyzed by using Materials Studio software (V 6.0, Accelrys, USA) based on intensity and width of the standard peak of crystalline Fe_3_O_4_ phase compared to the background peaks. The average crystallite size of MPs was calculated with the Debye–Scherrer equation:

$$D=\frac{0.94\times \lambda }{B\times \cos\theta }$$ (4)

Where D was the average crystallite size of MPs (nm), *λ* was the wavelength of the X-ray source (nm), *B* was the full width at half maximum (FWHM) of an individual peak (rad), and *θ* was the diffraction angle (^o^).

Compositions of MPs were determined by X-ray fluorescence (ARLADVANT XP^+^, Thermo, USA). The chemical state information on the particle surface was determined with an X-ray photoelectron spectrometer (ADES-400, VG, U.K.) using non-monochromated Mg Kα X-ray source. Particle morphologies were observed through a field emission scanning electron microscope (FE-SEM, FEI Co., USA) using a working voltage of 200 kV. The Brunauer-Emmett-Teller (BET) surface area of MPs was determined through nitrogen adsorption-desorption measurements (TriStar 3000, Micromeritics, USA). The Fourier transform infrared spectra were determined by a spectrometer (Nicolet 6700, Thermo, USA) using KBr wafer in the wavenumber range of 4000 cm^-1^ to 400^−1^. The zeta potential of MPs was measured by zeta potential analyzer (mastersizer 3000E, Malvern UK). Before analysis, 0.1 g MPs was suspended in 50 mL of KCl solution with concentration of 0.01 mol/L. pH of the suspension was adjusted from 1.4 to 10.7 by adding 1 M HCl or 1 M NaOH solutions, and agitated for 24 h. Then 1 mL sample was taken out for zeta potential analysis.

## Results and discussion

### Iron mud and MP composition

**Fig 1:**
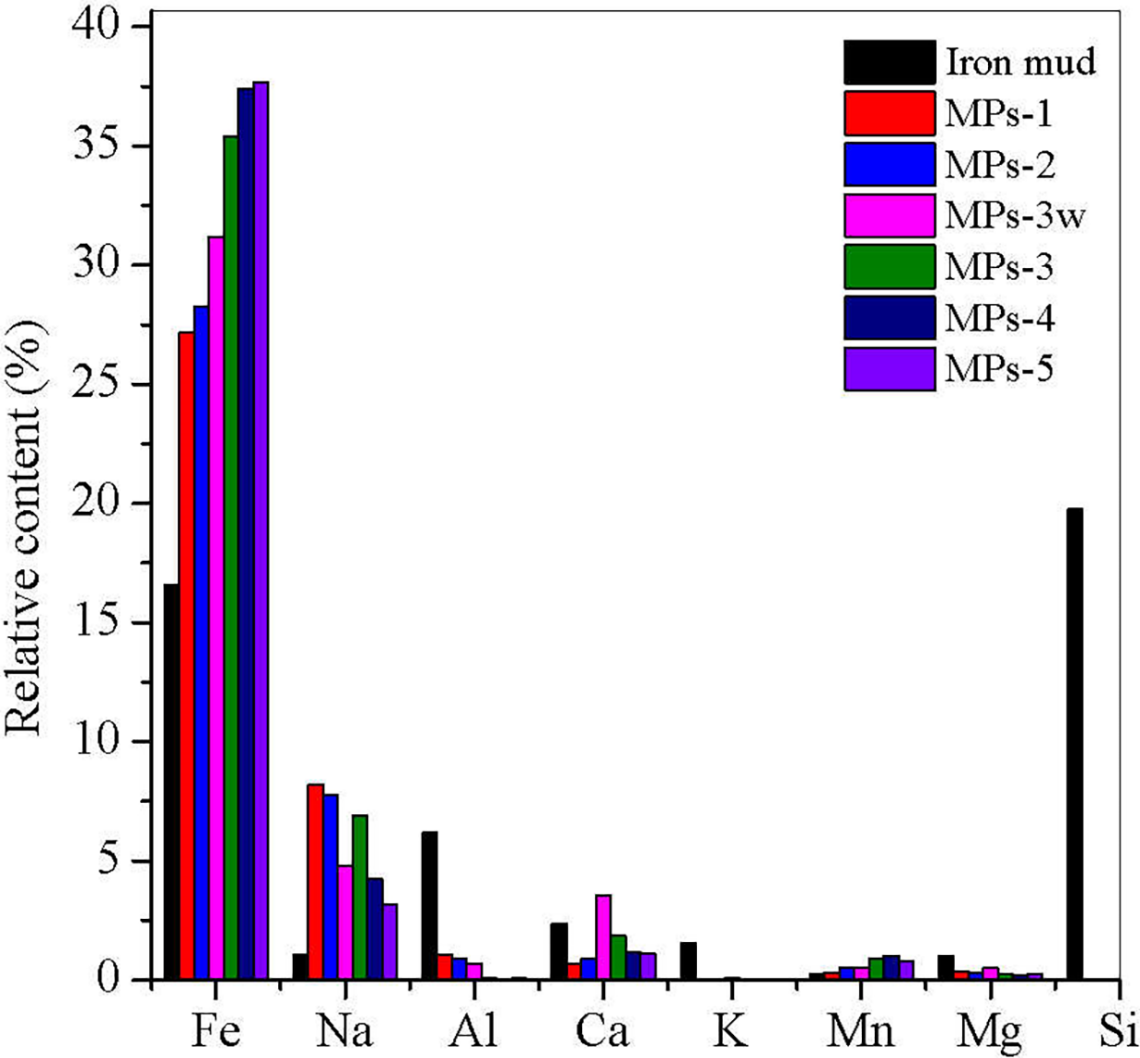
Major elements in iron mud and MPs. MPs 1–5 were synthesized by changing the molar ratio of ascorbic acid to Fe^3+^ from 0.01 to 0.05, 0.1, 0.15 and 0.2, respectively. MPs-3w was prepared at the molar ratio of 0.1 with acid wastewater digestion.

As demonstrated in Fig 1, the major elements of iron mud waste were Ca (2.4%), Al (6.2%), Fe (16.6%) and Si (19.8%) but Fe was dominant in the synthesized MPs (Fig 1). When iron mud was treated with nitric acid/acid wastewater, Fe and some metals leached out from mud into the liquid fraction, leaving some other elements mostly in the solid fraction, such as Mg, K, Ca, Al and Si. After separation from the solid fraction, Si was totally removed from, while Fe in the liquid fraction was re-precipitated to Fe_3_O_4_ under alkaline conditions and its content gradually increased when more H_2_A was added from MPs-1 to MPs-5 (Fig 1), indicating the crystallization of Fe_3_O_4_ increased with more available Fe^2+^. However, Na^+^ decreased from MPs-1 to MPs-5 because sizes of MPs increased with the increased crystallization, which led to the smaller surface area to support the functional groups (which will be discussed in the following sections). In the coprecipitation process, Na^+^ served as a ligand and was adsorbed onto the MP’s surface. The poorly crystalline iron oxide had a larger surface area, which support more functional groups for Na^+^ adsorption [21]. Large quantity of Ca was present in the acid wastewater (S1 Table), which increased Ca content in MPs-3w and negatively affected the adsorption.

### Mechanism of Fe_3_O_4_ synthesis

After H_2_A was added in the liquid fraction, Fenton reaction occurred in the presence of dissolved oxygen and Fe^3+^. Basically, H_2_A was firstly converted to ascorbate (HA^-^) after losing protons at its 2- or 3- positions, which were then replaced by Fe^3+^ ions [22, 23]. The attached Fe^3+^ ions were rapidly reduced to Fe^2+^, while HA^-^ was oxidized to *HA*∙ [24]. Finally, the formed *HA*∙ was captured by dissolved oxygen and quickly oxidized to dehydroascorbic acid (DHA) [23, 25] with formation of H_2_O_2_. The reactions were enhanced when the solution was exposed to the atmosphere. Other metal ions, such as Mn^2+^, Cu^2+^ and Zn^2+^, worked in a similar way in catalytic oxidation of H_2_A [26]. The resulting Fe^2+^ and other reduced ions, were re-oxidized by hydrogen peroxide to complete the cycle to form Fe^3+^ and other oxidized ions. Equations of the reactions were described as follow:

$$H_{2}A↔HA+H^{+}$$ (5)

$$HA^{‑}+Fe^{3+}\to {\left[HAFe\right]}^{2+}$$ (6)

$${\left[HAFe\right]}^{2+}\to {\left[FeHA•\right]}^{2+}\to HA•+Fe^{2+}$$ (7)

$$HA•+O_{2}\to HAO_{2}•$$ (8)

$$HAO_{2}•+H^{+}\to DHA+H_{2}O_{2}$$ (9)

$$H_{2}O_{2}+Fe^{2+}\to Fe^{3+}+OH+•OH$$ (10)

$$Fe^{2+}+•OH\to Fe^{3+}+OH^{‑}$$ (11)

DHA was unstable and easily hydrolyzed to _L_-diketogulonate (2,3-DKG) at pH 7 [24, 27]. In the presence of high concentration of H_2_O_2_, both DHA and 2,3-DKG was oxidized and broken down rapidly to _L_-threonate and oxalate [28].

$$DHA+H_{2}O\to 2,3‑DKG$$ (12)

$$DHA+H_{2}O_{2}\to L‑threonate+oxalate$$ (13)

$$2,3‑DKG+H_{2}O_{2}\to L‑threonate+oxalate$$ (14)

After the dissolved oxygen in the liquid fraction exhausted, H_2_O_2_ was not produced. Therefore, stable Fe^2+^ was formed by continuous reduction of Fe^3+^ with H_2_A. Fe^2+^ tended to coprecipitate with Fe^3+^ when the two types of ions were present under high pH (Eq 15), which resulted in formation of Fe_3_O_4_ particles.

$$Fe^{2+}+2Fe^{3+}+8OH^{‑}\to Fe{\left(OH\right)}_{8}\to Fe_{3}O_{4}+4H_{2}O$$ (15)

### Magnetization measurement

**Fig 2:**
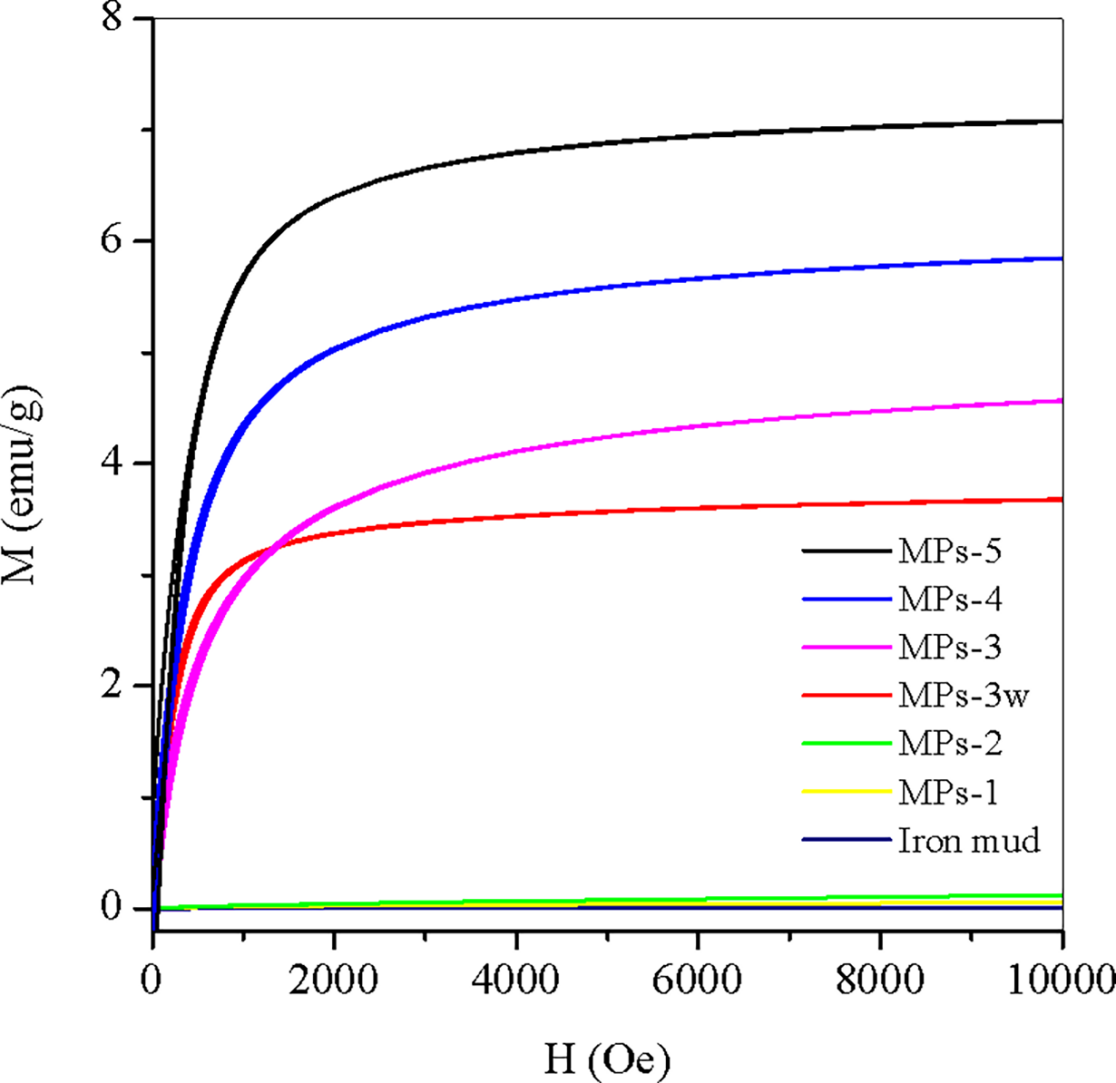
Magnetic hysteresis loops of iron mud and MPs. MPs 1–5 were synthesized by changing the molar ratio of ascorbic acid to Fe^3+^ from 0.01 to 0.05, 0.1, 0.15 and 0.2, respectively. MPs-3w was prepared at the molar ratio of 0.1 with acid wastewater digestion.

As shown in Fig 2, MPs-3, MPs-4, MPs-5M and MPs-3w demonstrated strong magnetism with low remanence and coercivity, indicating these MPs had soft magnetism and easily removed from the treated water by simply placing a magnetic field after MB adsorption (see graph abstract). By increasing the H_2_A/Fe^3+^ ratio, the saturation magnetization (Ms) increased from 3.7, 4.6, 5.9 to 7.1 emu/g for MPs-3w, MPs-3, MPs-4 and MPs-5, respectively, which indicated magnetic response became stronger with increasing Fe^2+^/Fe^3+^ ratio. Similar to iron mud, MPs-1 and MPs-2 were synthesized with lower H_2_A/Fe^3+^ ratios, demonstrated weak magnetic response, and would not be considered in the following studies.

**Fig 3:**
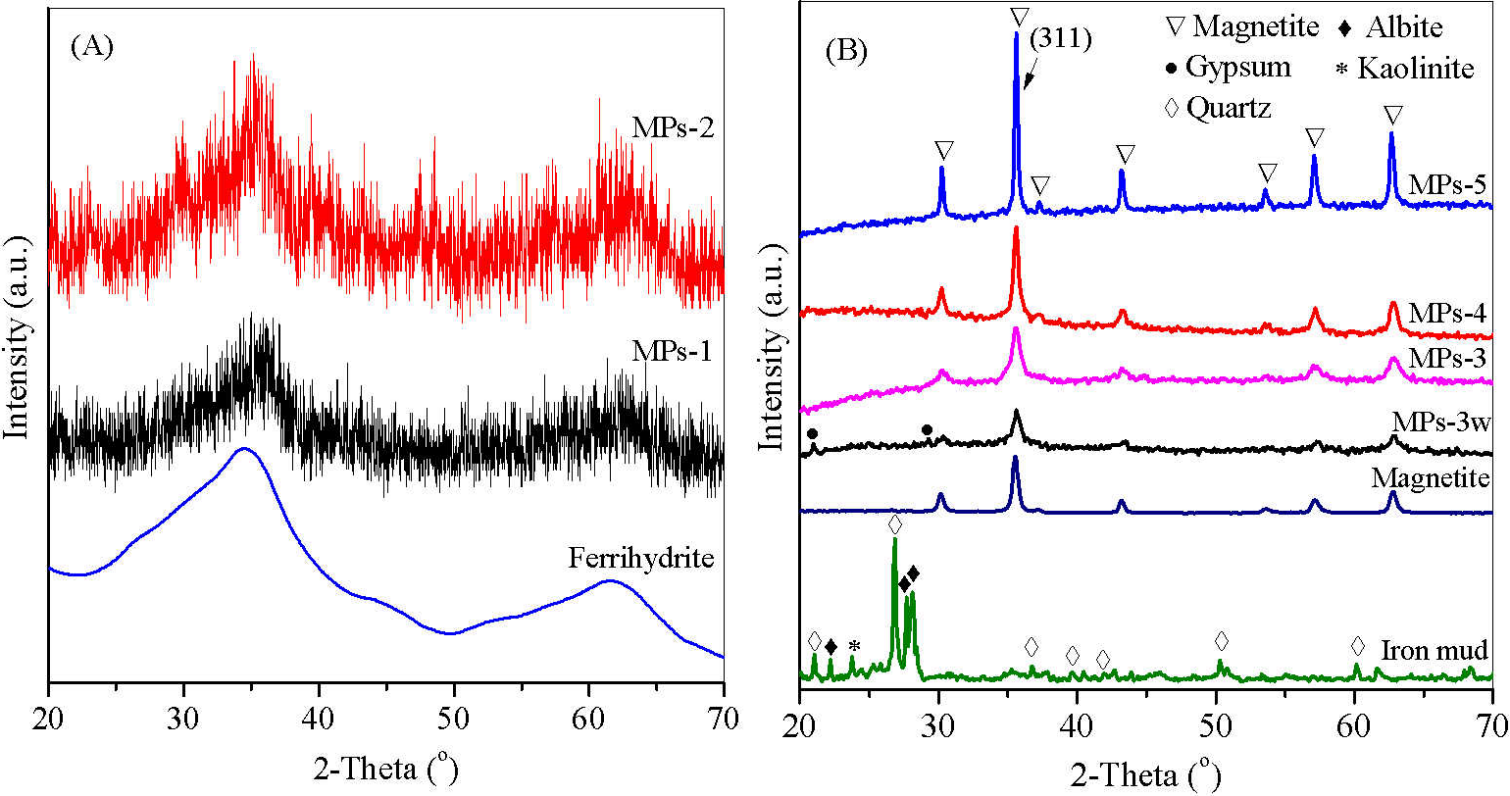
**XRD pattern of (A) MPs-1, MPs-2 and ferrihydrite, and (B) MPs-3w, MPs-3, MPs-4, MPs-5 and Iron mud.** MPs 1–5 were synthesized by changing the molar ratio of ascorbic acid to Fe^3+^ from 0.01 to 0.05, 0.1, 0.15 and 0.2. MPs-3w was prepared at the ratio of 0.1 with acid wastewater digestion.

In precipitation of Fe_3_O_4_, it nucleated when Fe^2+^ concentration reached the critical supersaturation level, and the nuclei grew by diffusing Fe^2+^ to the surface of Fe_3_O_4_ [29]. When the H_2_A/Fe^3+^ ratio was smaller than 0.05, the limited H_2_A^-^ was completely exhausted by dissolved oxygen and other cations, such as Mn^4+^ [15, 30], resulting in inadequate Fe^2+^ accumulation for nucleation of ferrihydrite (Fig 3A). The strongest Ms was generated when the Fe^2+^/Fe^3+^ ratio was between 0.67 and 1 in MPs coprecipitation and the small Fe^2+^/Fe^3+^ ratio led to a weak magnetic response [31]. The magnetic response of Fe_3_O_4_ was size-dependent. When the Fe^2+^/Fe^3+^ ratio increased, the size of Fe_3_O_4_ particles increased [32] and the magnetic response increased correspondingly (Fig 2). The large size of Fe_3_O_4_ particles reduced the surface to volume ratio, which may decrease the thermal fluctuation and magnetic disorder of Fe_3_O_4_ molecules, leading to strong magnetic response [33].

A acid wastewater with a pH of 0.61 from a propylene plant, which mainly contained acid species of Cl^-^ (3992.5mg/L), NO_3_^-^ (444.4mg/L) and SO_4_^2-^ (22492.2mg/L) (S1 Table), was tested to replace the commercial nitric acid in dissolving iron mud for Fe_3_O_4_ production. The wastewater also contained 185.9 mg/L of Fe^3+^, which could contribute to iron species in MP synthesis. However, significant amount of Ca^2+^ (3736.1 mg/L) and other impurities of ion species may interrupt the reactions (S1 Table). The synthesized MPs using acid wastewater as a dissolving reagent (MPs-3w) demonstrated an Ms of 3.7 emu/g, slightly lower than 4.6 emu/g of MPs-3 (Fig 2), probably because the generated gypsum by Ca^2+^ and SO_4_^2-^ precipitation negatively affected the unit magnetite of Fe_3_O_4_. It was reported that Ms was greatly reduced to half from 57.8 emu/g to 25 emu/g when Fe_3_O_4_ particles were coated by Zr(SO_4_)_2_ [34]. However, it was significant that the commercial nitric acid could be replaced by the negatively valued wastewater to reduce the cost of Fe_3_O_4_ production. Currently, studies are being carried out to synthesize Fe_3_O_4_ with the reduced gypsum in acid wastewater.

### XRD analysis

XRD was used to characterize the crystallography of the obtained MPs. The XRD patterns of iron mud and the synthesized MPs were shown in Fig 3A and 3B, respectively with controls of ferrihydrite and magnetite. The main peaks corresponding to *2θ* of 34^o^ and 61^o^ in MPs-1 and MPs-2 were related to the poorly crystallized ferrihydrite (Fig 3A) [35]. For iron mud, the impurities, such as quartz and albite, were reflected by the intensities of diffraction peaks, which covered the main peak of the poor crystallized iron oxide at *2θ* of 34^o^ (Fig 3B). The reflection peaks at *2θ* of 30.1^o^, 35.4^o^, 43.2^o^, 53.4^o^, 57.2^o^ and 62.7^o^, attributing to diffractions from the planes of (220), (311), (400), (422), (511), (440) of magnetite Fe_3_O_4_ (JCPDS 76–1849) with cubic spinel structure, were observed in the synthesized MPs-3w, MPs 3–5, and the magnetite control (Fig 3B), indicating Fe_3_O_4_ was present in MPs. In addition, the peaks observed in MPs-3w at *2θ* = 20.9° and 29.3° were affiliated to gypsum (JCPDS 33–0311) [36], which was due to CaSO_4_ precipitation at pH 9.5 because of high concentrations of Ca^2+^ and SO_4_^2-^ in the acid wastewater (S1 Table).

The crystallite sizes were calculated by using FWHM of the strongest peak (311), which were 3.16, 4.35, 7.56 and 18.32 nm for MPs-3w and MPs 3–5, respectively. The results demonstrated that by increasing the H_2_A/Fe^3+^ ratio, the average sizes of MP crystallite increased accordingly. The result was consistent with the studies of Samaneh et al., in which the average crystallite size of magnetite increased almost double from 9.07 to 18.97 nm when the Fe^2+^/Fe^3+^ ratio was increased from 0.5 to 2 [32]. In preparing MPs-3w, acid wastewater containing Fe^3+^ replaced nitric acid and contributed iron species in Fe_3_O_4_ synthesis. The combined Fe^3+^ for MPs-3w led to a lower Fe^2+^/Fe^3+^ ratio and therefore a smaller particle size of 3.16 nm.

It was demonstrated that amorphous iron oxide had higher surface area than the crystallized one (Fig 3), which exposed more ion-exchange functional groups on the surface [37, 38]. Among the selected MPs in MB adsorption, MPs-3 was least crystallized and had the largest surface area as demonstrated by the BET analysis in the following N_2_ adsorption isotherms, which contained the most functional groups and the highest adsorption capacity. When the H_2_A/Fe^3+^ ratio increased steadily from MPs-3 to MPs-5, the synthesized MPs became more crystallized, reducing the surface area and functional groups on it, which was consistent with the reduced MB adsorption capacity shown in the following isothermal equilibrium. This result was consistent with the observation of Zhang et al. that amorphous Fe_3_O_4_ showed better adsorption capacity to Pb(II) and Cd(II) than the well crystallized α- and γ- Fe_2_O_3_ [37]. It was reported that after ferrihydrite was converted to more crystallized goethite, its adsorption capacity to radium decreased from nearly 100% to 20% [39].

### XPS analysis

**Fig 4:**
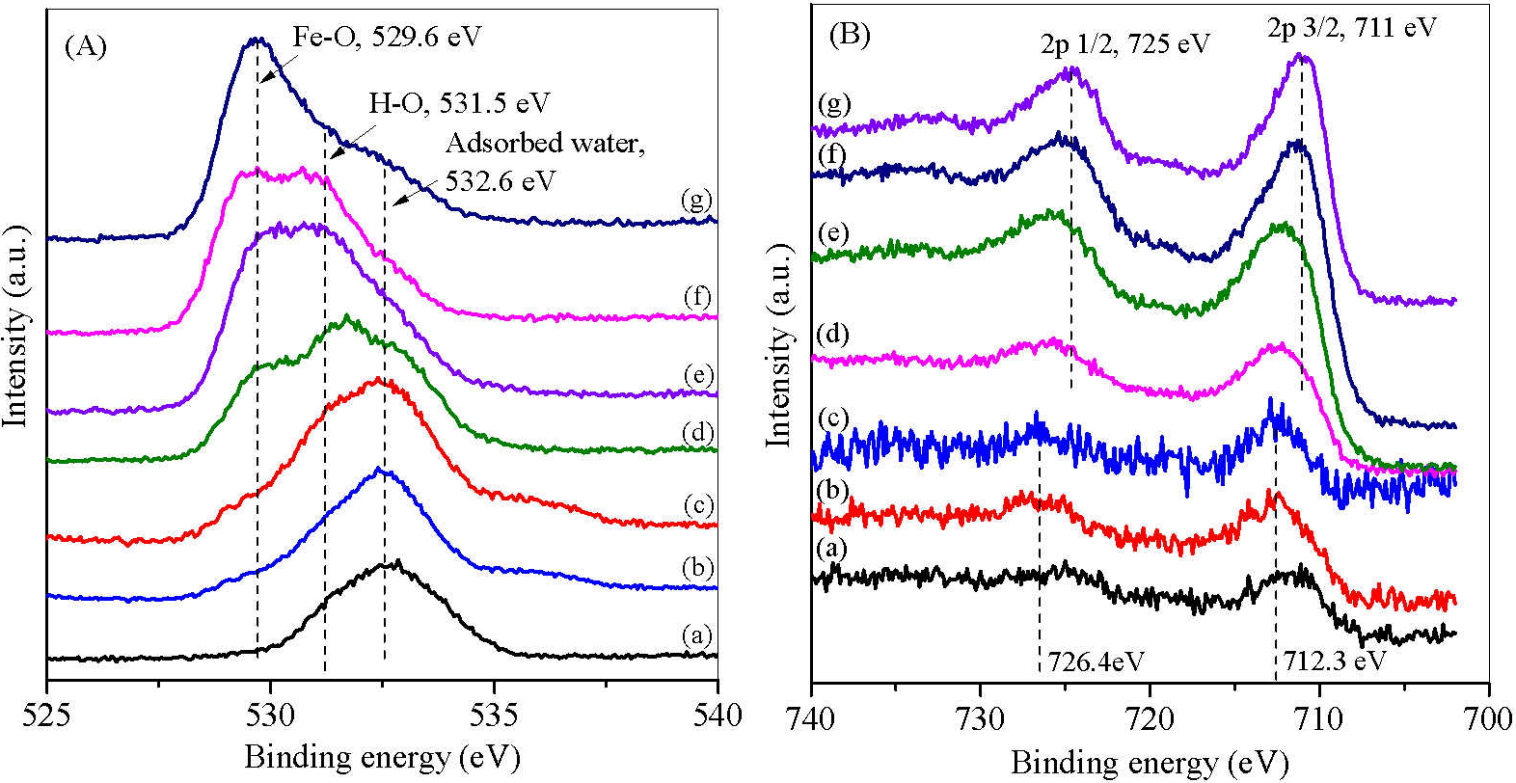
**XPS O1s (A) and Fe2p (B) core-level spectra of iron mud (a) and MPs-1 (b), MPs-2 (c), MPs-3w (d), MPs-3 (e), MPs-4 (f) and MPs-5 (g).** MPs 1–5 were synthesized by changing the molar ratio of ascorbic acid to Fe^3+^ from 0.01 to 0.05, 0.1, 0.15 and 0.2. MPs-3w was prepared at the ratio of 0.1 with acid wastewater digestion.

To further investigate the valence states of Fe and O in iron mud and MPs, O1s and Fe2p spectra of XPS were analyzed and the two spectra demonstrated consistent results. In O1s spectrum, the Fe-O peak at 529.6 eV was observed in MPs-3w and MPs-3 to 5, indicating the formation of Fe_3_O_4_ [40]. The peak at 712.3 eV and 726.4 eV was shown in Fe2p spectrum of MPs 1–2, representing Fe 2p_3/2_ and Fe 2p_1/2_ of ferric species in ferrihydrite (Fig 4B) [41]. By increasing the HA/Fe^3+^ ratios, Fe 2p_3/2_ and Fe 2p_1/2_ shifted to 711 eV and 725 eV, respectively for MPs 3–5, corresponding the increased Fe^2+^/Fe^3+^ ratios in MPs. The results indicated that Fe_3_O_4_ content increased by increasing HA/Fe^3+^ ratios, which corresponded to the increased magnetic response from MPs-1 to MPs-5 demonstrated in Fig 2A.

### SEM analysis

**Fig 5:**
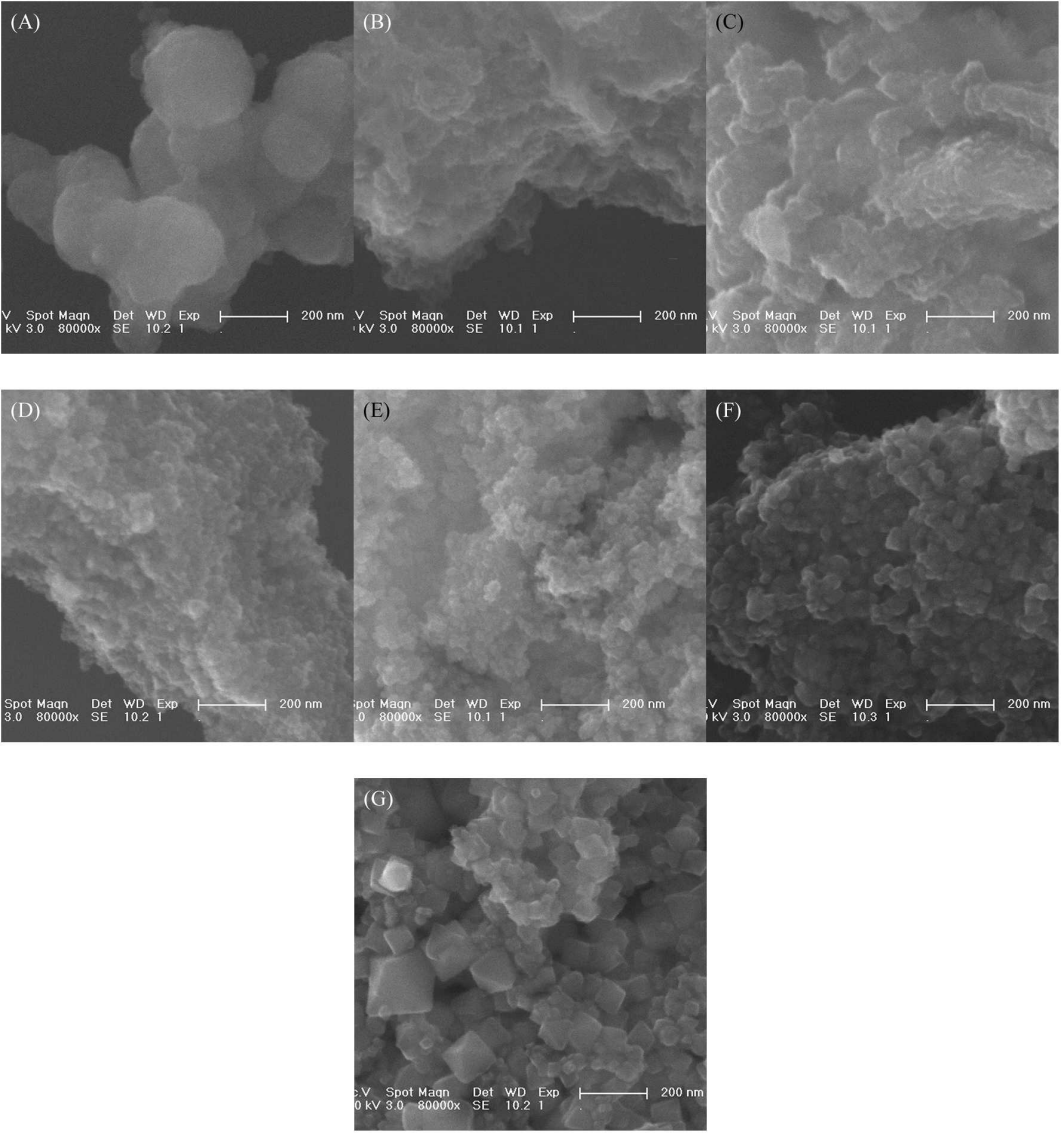
**SEM images of iron mud (A), MPs-1 (B), MPs-2 (C), MPs-3w(D), MPs-3 (E), MPs-4(F) and MPs-5(G).** MPs 1–5 were synthesized by changing the molar ratio of ascorbic acid to Fe^3+^ from 0.01 to 0.05, 0.1, 0.15 and 0.2. MPs-3w was prepared at the ratio of 0.1 with acid wastewater digestion.

The morphology of iron mud and MPs was characterized by SEM. As shown in Fig 5A, iron mud was about 200 nm particles with smooth surface. The morphologies of MPs-1 and MPs-2 were irregular because of the formation of amorphous ferrihydrite (Fig 5B and 5C). When more H_2_A was added, more Fe^2+^ was generated, which was favorable to the nuclei growth of Fe_3_O_4_ and led to bigger size of MPs (Fig 5D–5G). The result was consistent with the XRD spectrum and magnetic response.

### Sorption of MB

#### Sorption kinetics

**Fig 6:**
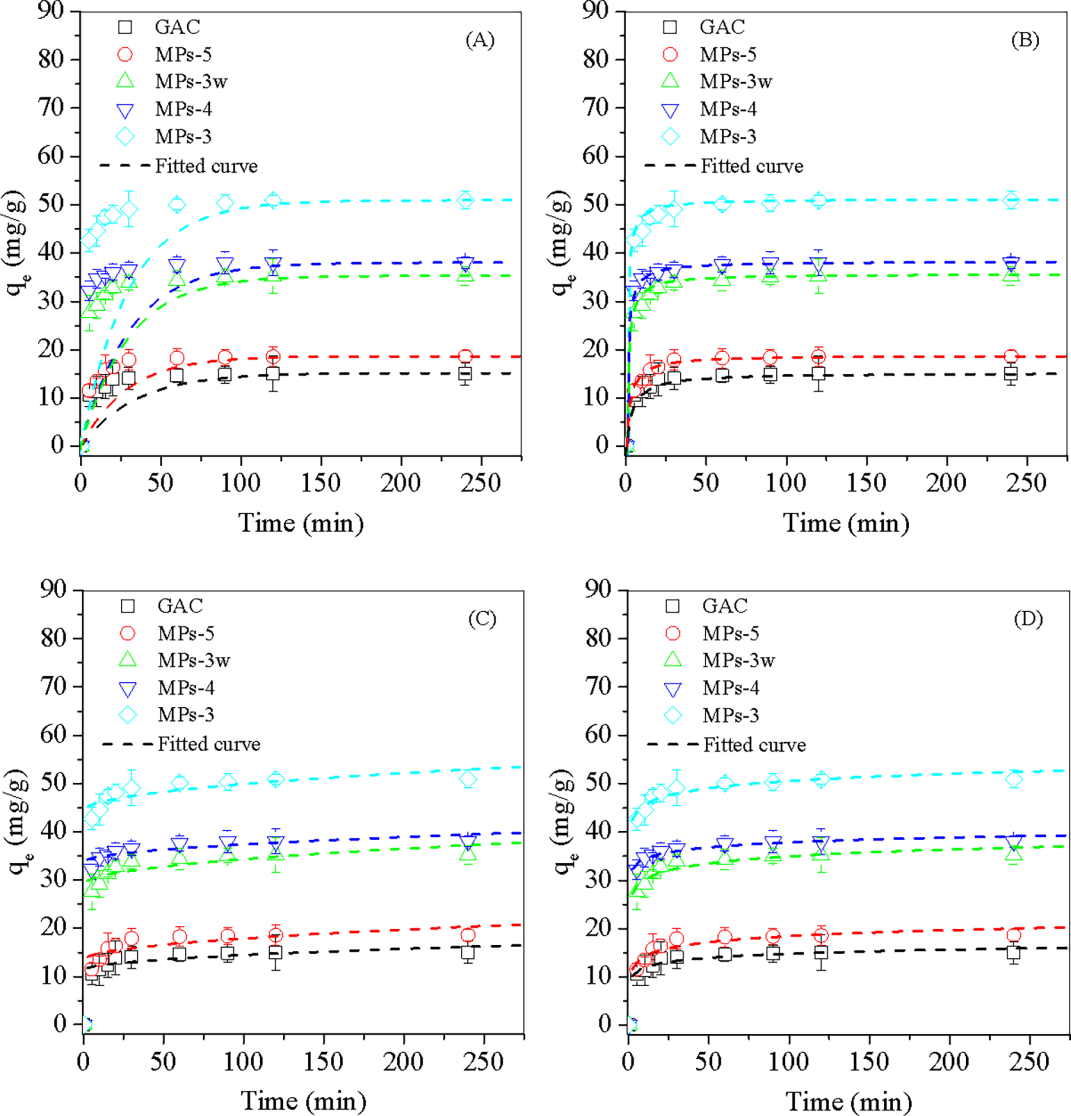
**Nonlinear Fitting curve of pseudo-first-order kinetic (A), pseudo-second-order kinetic (B), inter-particle diffusion (C) and Elovich kinetic (D) for MB adsorption on MPs and GAC.** Herein, MPs 3–5 were synthesized by changing the molar ratio of ascorbic acid to Fe^3+^ from 0.1 to 0.15 and 0.2, respectively. MPs-3w was prepared at the molar ratio of 0.1 with acid wastewater digestion.

MPs-3w and MPs 3–5 had good magnetic response, and were further studied for MB adsorption, in which the widely used MB adsorbent of GAC was a reference. Time-course of MB adsorption on MPs and GAC were investigated as shown in Fig 6. MPs and GAC showed similar adsorption trend. The adsorption capacity increased rapidly at the initial 30 min, and slowly in the following 90 min, and kept constant within the subsequent 120 min, indicating the adsorption was equilibrated in 120 min. The kinetics of adsorption of MB on MPs-3w, MPs 3–5 and GAC were studied as a function of adsorption time at 25 ^o^C. As shown in Fig 6, four typical kinetic models, such as Lagergren's pseudo-first order (Eq 16), pseudo-second order kinetics model (Eq 17), inter-particle diffusion model (Eq 18) and kinetic model (Eq 19) proposed by Elovich et al. [42], were tested for the experimental data. The four kinetics models were expressed as follows:

$$\ln(q_{e}-q_{t})=\ln q_{e}-k_{1}t$$ (16)

$$\frac{t}{q_{t}}=\frac{1}{k_{2}q_{e}^{2}}+\frac{t}{q_{e}}$$ (17)

$$q_{t}=k_{i}t^{0.5}+C$$ (18)

$$q_{t}=\frac{1}{\beta }\ln(\alpha \beta )+\frac{1}{\beta }\ln(t)$$ (19)

Where, *q*_*e*_ and *q*_*t*_ are the equilibrium adsorption capacity (mg/L) and the adsorption capacity (mg/L) at t min, respectively. *k*_*1*_ is the pseudo-first-order model rate constant (min^-1^). In Eq (17), *k*_*2*_ is the pseudo-second-order adsorption rate constant (g/mg.min). In Eq (18), *k*_*i*_ is the intra-particle diffusion constant (mg/g. min^1/2^). In Eq (19), α and β is the initial adsorption rate (g/mg.min) and desorption constant (mg/g.min), separately.

The parameters obtained from the four kinetic models are in Table 1, which showed the pseudo-second-order model had the highest R^2^ values in the four kinetics models. All R^2^ values of pseudo-second-order model were larger than 0.999, suggested that pseudo-second-order model was the best fit for the experimental data, indicating. MPs-3 exhibited the highest *k*_*2*_ value of 0.0254 g/mg. min than other MPs and GAC, indicated that MPs-3 has the highest adsorption rate.

**Table 1:** Parameters and the regression coefficients (R^2^) of the kinetic models.

**Kinetic model**	**Parameters**	**MPs-3**	**MPs-4**	**MPs-5**	**MPs-3w**	**GAC**
	*q*_e,exp_ (mg/g)	50.95	38.04	18.66	35.32	15.17
**Pseudo-first-** **order**	*k*_*1*_ (/min)	0.034	0.033	0.035	0.034	0.03
	*R*^*2*^	0.936	0.972	0.897	0.938	0.933
**Pseudo-second-order**	*q*_e,cal_ (mg/g)	51.23	38.27	18.85	35.69	15.2
	*k*_*2*_ (g/mg. min)	0.0254	0.0242	0.0198	0.0217	0.0156
	*R*^*2*^	0.9992	0.9999	0.9999	0.9995	0.9997
**Intra-particle diffusion**	*C*	44.43	33.68	13.43	29.08	11.33
	*k*_*i*_ (mg/g. min^1/2^)	0.547	0.371	0.446	0.524	0.312
	*R*^*2*^	0.6157	0.6253	0.5274	0.6055	0.6039
**Elovich equation**	*Α*	3.67×10^8^	2.74×10^9^	512	476007	2226
	*Β*	0.466	0.686	0.556	0.485	0.818
	*R*^*2*^	0.8523	0.8655	0.7863	0.8443	0.8352

#### Isothermal equilibrium study

To further investigate the sorption mechanism, the sorption isotherms of MB on MPs and GAC were measured, respectively. Six of the most established isotherm models, such as langmuir isotherm (Eq 20), Freundlich isotherm (Eq 21), Redlich-Peterson isotherm (Eq 22), Tempkin isotherm (Eq 23), Sips isotherm (Eq 24) and Toth isoisotherm (Eq 25), were used to test the experimental data and the equations were expressed as follows:

$$\frac{C_{e}}{q_{e}}=\frac{1}{K_{L}}+\frac{a_{L}}{K_{L}}C_{e}$$ (20)

$$\ln q_{e}=\ln K_{F}+b_{F}\ln C_{e}$$ (21)

$$\frac{C^{e}}{q^{e}}=\frac{1}{K_{R}}+\frac{a_{R}}{K_{R}}C_{e}^{\beta }$$ (22)

$$q_{e}=B\ln A+B\ln C_{e}$$ (23)

$$q_{e}=\frac{q_{m}{\left(b_{S}C_{e}\right)}^{\frac{1}{n}}}{1+{\left(b_{S}C_{e}\right)}^{\frac{1}{n}}}$$ (24)

$$q_{e}=\frac{K_{T}C_{e}}{{\left(a_{T}+C_{e}^{t}\right)}^{\frac{1}{t}}}$$ (25)

Where, *K*_*L*_ and *a*_*L*_ were the Langmuir isotherm constants; *K*_*F*_ and *b*_*F*_ were the Freundlich constants; *K*_*R*_, *a*_*R*_ and *β* were the Redlich-Peterson isotherm constants; *A* and *B* were the Tempkin isotherm constant; *b*_*S*_ and *1/n* were the Sips isotherm constant; *K*_*T*_, *a*_*T*_ and *t* were the Toth isotherm constant.

**Fig 7:**
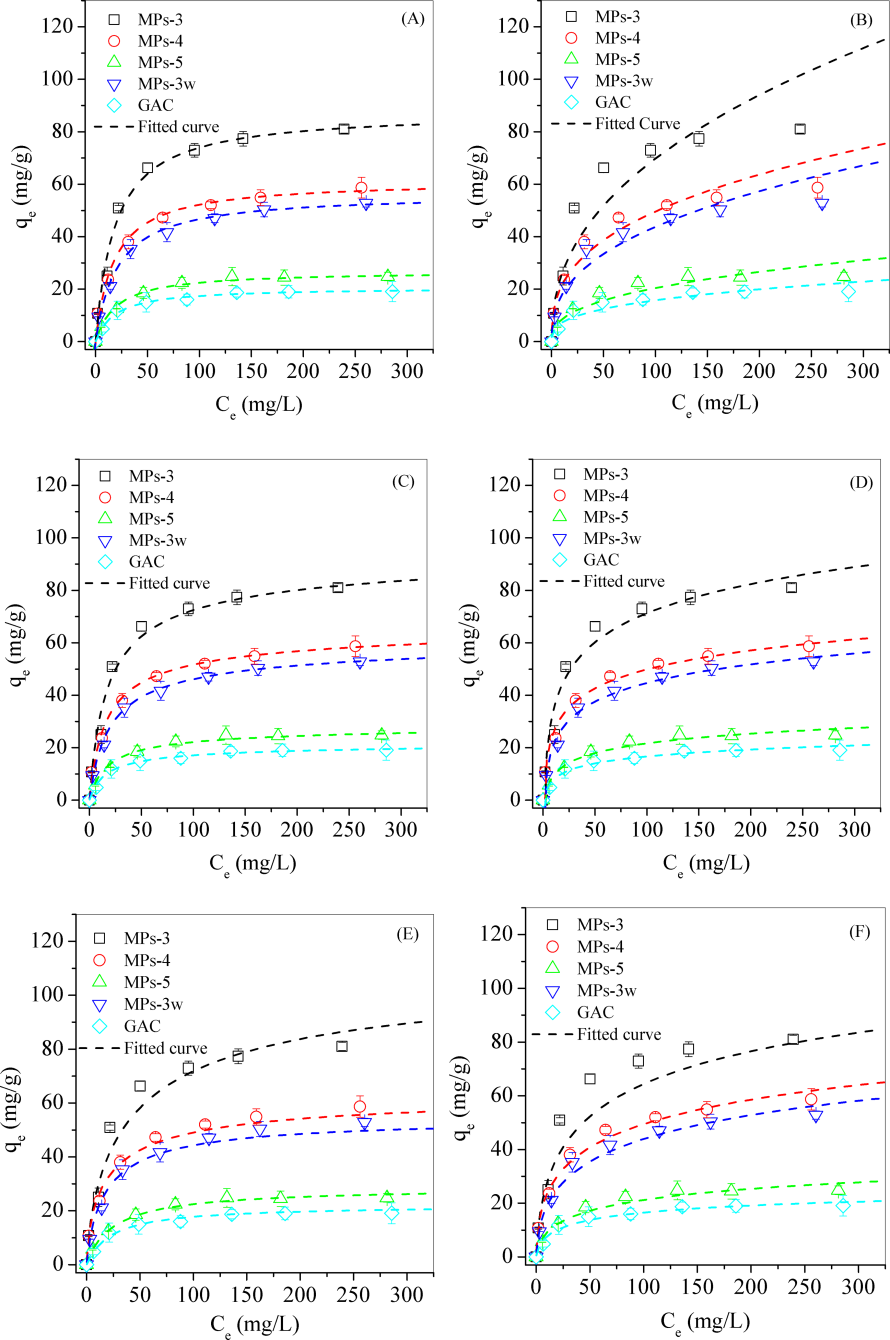
**Fitting curve of Langmuir (A), Freundlich (B), Redlich-Peterson (C), Tempkin (D), Sips (E) and Toth (F) isotherm for MB adsorption on MPs and GAC.** Herein, MPs 3–5 was synthesized by changing the molar ratio of ascorbic acid to Fe^3+^ from 0.1 to 0.15, 0.2. MPs-3w was prepared at the molar ratio of 0.1 with acid wastewater digestion.

The non-linear fitting curves of the six isotherm models were shown in Fig 7, and the isotherm parameters of each model were calculated with the correlation coefficient (R^2^) as shown in S2 Table, in which the Langmuir and Redlich-Peterson isotherms fitted better for the experimental data than Freundlich, Templin, Sips and Toth isotherms. However, the constant *β* from the Redlich-Peterson isotherm was 0.95, closed to 1. When the constant *β* = 1, the equation of the Redlich-Peterson isotherm was reduced to the Langmuir isotherm [43]. Thus, it was concluded that the Langmuir isotherm provided the best description of the experimental data.

The Langmuir isotherm model was based on an idealized assumption of identical sorption heat and monolayer sorption. The calculated results from Langmuir isotherm showed that all MPs had higher capacity in MB adsorption than GAC (Fig 7A). Among MPs, the order for MB adsorption was MPs-3 > MPs-4 > MPs-3w > MPs-5 and the best adsorbent of MPs-3 had a capacity of MB adsorption 3.8 times of GAC. Because acid wastewater contained high content of Ca^2+^ (3736.1 mg/L) (S1 Table), gypsum phase was formed in MPs-3w, which negatively affected MB adsorption (Fig 7A). The adsorption isotherms was converted to Langmuir isotherm model for calculating adsorption capacity *q*_*m*_ (Fig 7B). MPs-3 demonstrated the highest capacity of 87.3 mg/g in MB adsorption, which was significantly higher than 61.8 mg/g and 56.7 mg/g of MPs-4 and MPs-3w, respectively. MPs-5 showed the lowest capacity of 27.4 mg/g. The highest MB adsorption capacity of MPs-3 may be related to its largest surface area, to which most functional groups were attached. With the increased HA/Fe^3+^ ratio from MPs-3 to MPs-5, the average particle sizes increased, which reduced the unit surface area and functional groups on it.

#### Regeneration and column experiments

**Fig 8:**
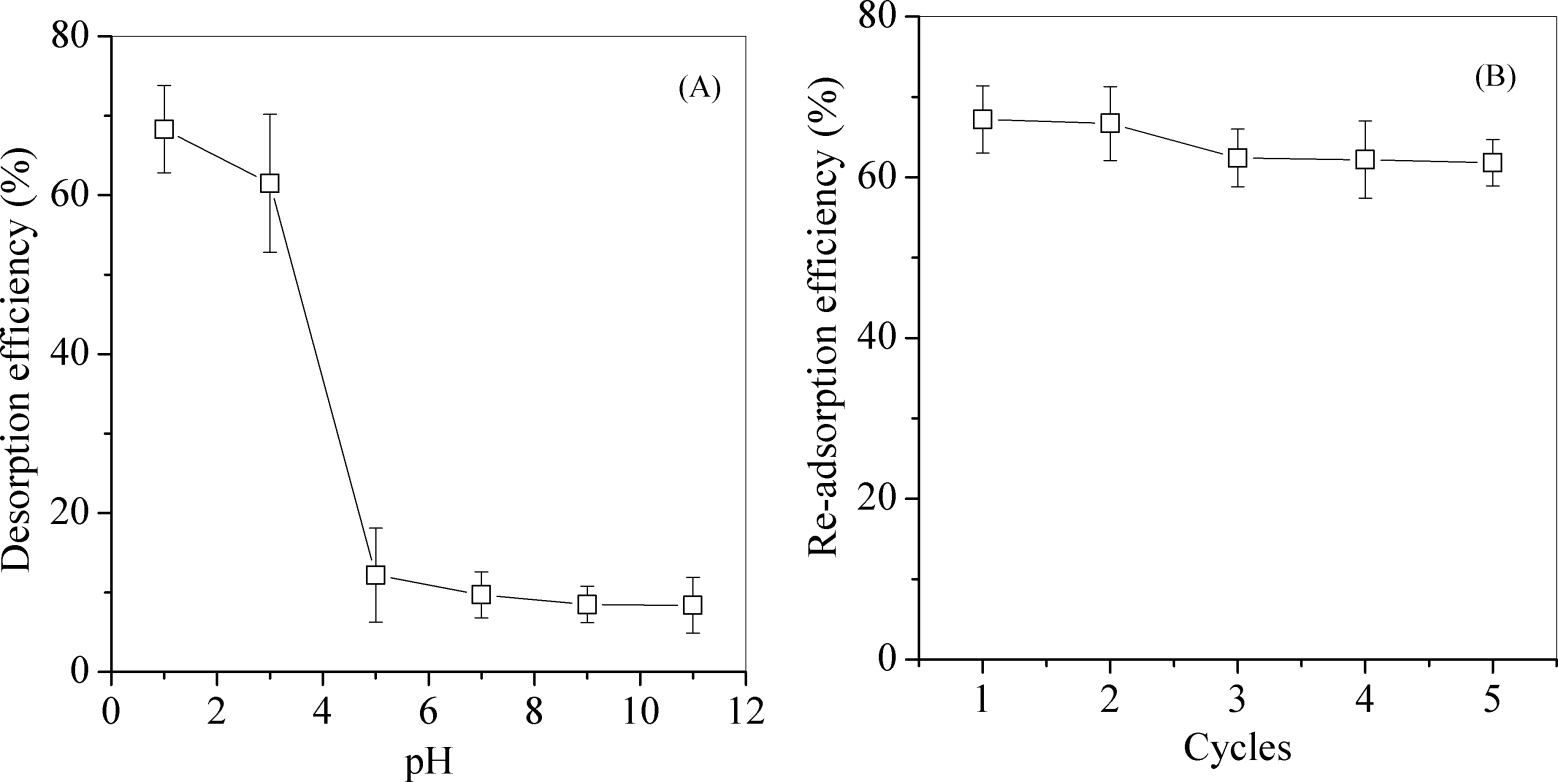
The desorption (A) and re-adsorption (B) efficiency of MPs-3 for MB removal.

MPs-3 had the highest adsorption capacity among the synthesized MPs and was used for regeneration experiments. The effect of pH in desorption was shown in Fig 8A. It was found that the MB desorption efficiency reduced with increase of pH and the best pH for desorption was 1 (Fig 8A), which indicated competition between H^+^ and MB under acidic conditions favored MPs desorption, and pH 1 was selected for desorption. As shown in Fig 8B, at the end of five cycles, the efficiency of MB adsorption was 62%, slightly decreased from 67% of the first round, indicating MP-3 could be repeatedly used in MB adsorption.

**Fig 9:**
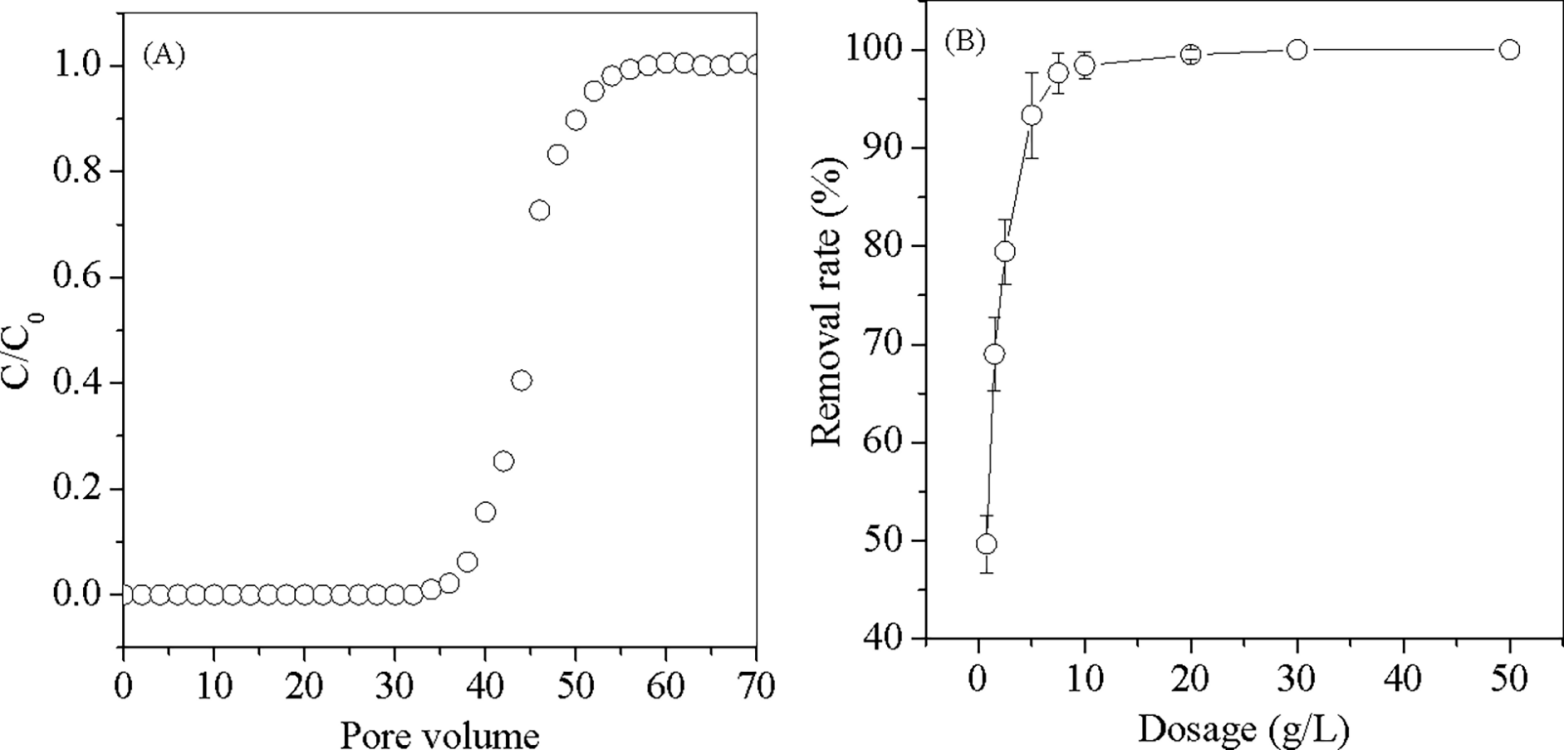
Breakthrough curve (A) and Removal rate (B) of MB adsorption by MPs-3.

Column experiment was carried out and the breakthrough curve of MB adsorbed on MPs-3 was shown in Fig 9A. MPs-3 exhibited effective adsorption ability, and completely adsorbed MB from the influent before the breakthrough point. Pilot experiments of MPs-3 adsorption for MB was carried out as shown in Fig 9B. 49.7% of MB was removed by adding 0.75 g/L of MPs-3, while only 22% of MB was removed by adding the same dose of GAC. When MPs-3 was increased to 10 g/L, 99% of MB was removed, which was consistent with the MB removal by MPs-3 at the bench scale, indicated that MPs-3 was effective in removing MB at the pilot scale.

To determine whether MPs-3 was practical in industrial application, a cost analysis was carried out in treating a synthesized dye wastewater containing MB. It was assumed to remove 99% of MB from 1 ton of wastewater containing 100 mg/L MB, and 10 kg of MPs-3 was used, which needed about US$ 6.54 for synthesis (S3 Table). It was much lower than the cost of GAC (US$ 52) to treat the same amount MB. In addition, MPs-3 could be repeatedly used after regeneration, which further reduced the cost for MPs-3 to treat MB-containing wastewater.

#### The mechanism of MB adsorption

**Fig 10:**
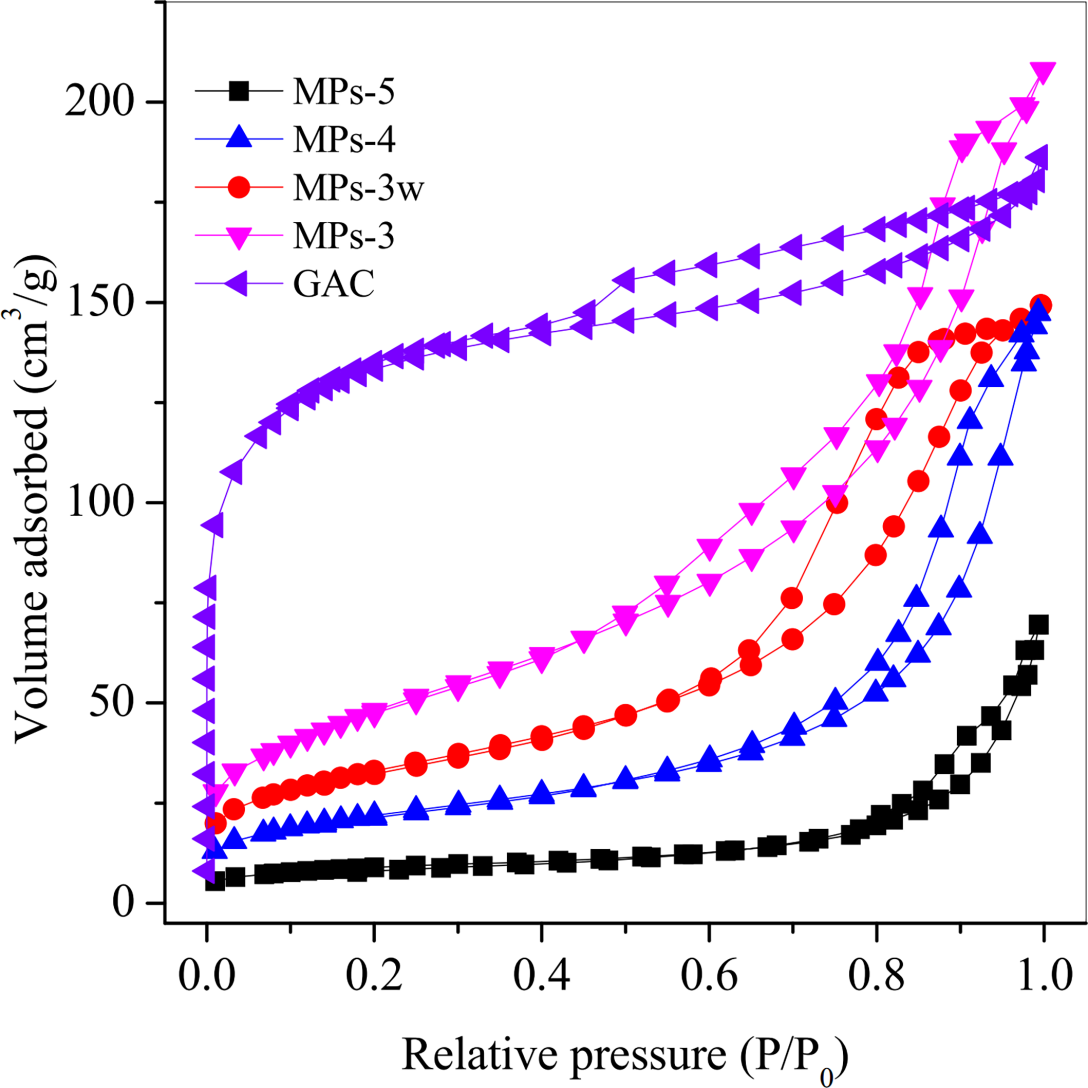
N_2_ adsorption isotherms of MPs and GAC. Herein, MPs 3–5 were synthesized by changing the molar ratio of ascorbic acid to Fe^3+^ from 0.1 to 0.15, 0.2, separately. MPs-3w was prepared at the molar ratio of 0.1 by acid wastewater digestion.

To investigate the effect of surface area on MB adsorption, N_2_ adsorption isotherms were made for MPs and GAC. It was demonstrated that GAC was much more favorable in adsorption of inert N_2_ gas than MPs, indicating GAC had much larger surface area than MPs (Fig 10). Indeed, the BET surface area for GAC was 442.8 m^2^/g, much larger than 33.6 m^2^/g, 79.1 m^2^/g, 118.9 m^2^/g and 176.2, m^2^/g for MPs-5, MPs-4, MPs-3w and MPs-3, respectively. The high surface area and low MB adsorption capacity of GAC indicated that the surface area was not playing primary role in MB adsorption. Among the MPs, the BET surface area decreased in the order of MPs-3 > MPs-4 > MPs-5, which was consistent with the MB adsorption capacity, indicating the Fe-O^-^ functional groups on Fe_3_O_4_ surface played a significant role in MB adsorption. The bigger the surface area, the more Fe-O^-^ functional groups it contained, and the better adsorption for MB. The cation exchange capacity (CEC) of MPs was further tested with NH_4_-Na methods. As expected, MPs-3 showed the highest CEC, followed by MPs-4 and MPs-5 (Table 2), which provided direct evidence that cation exchange was the mechanism for MB adsorption.

**Table 2:** Cation exchange capacity of MPs by NH_4_-Na test. (Unit: Mmol/kg).

cation	MPs-3w	MPs-3	MPs-4	MPs-5
**Na**^**+**^	840.12	1378.76	1085.13	271.4

**Fig 11:**
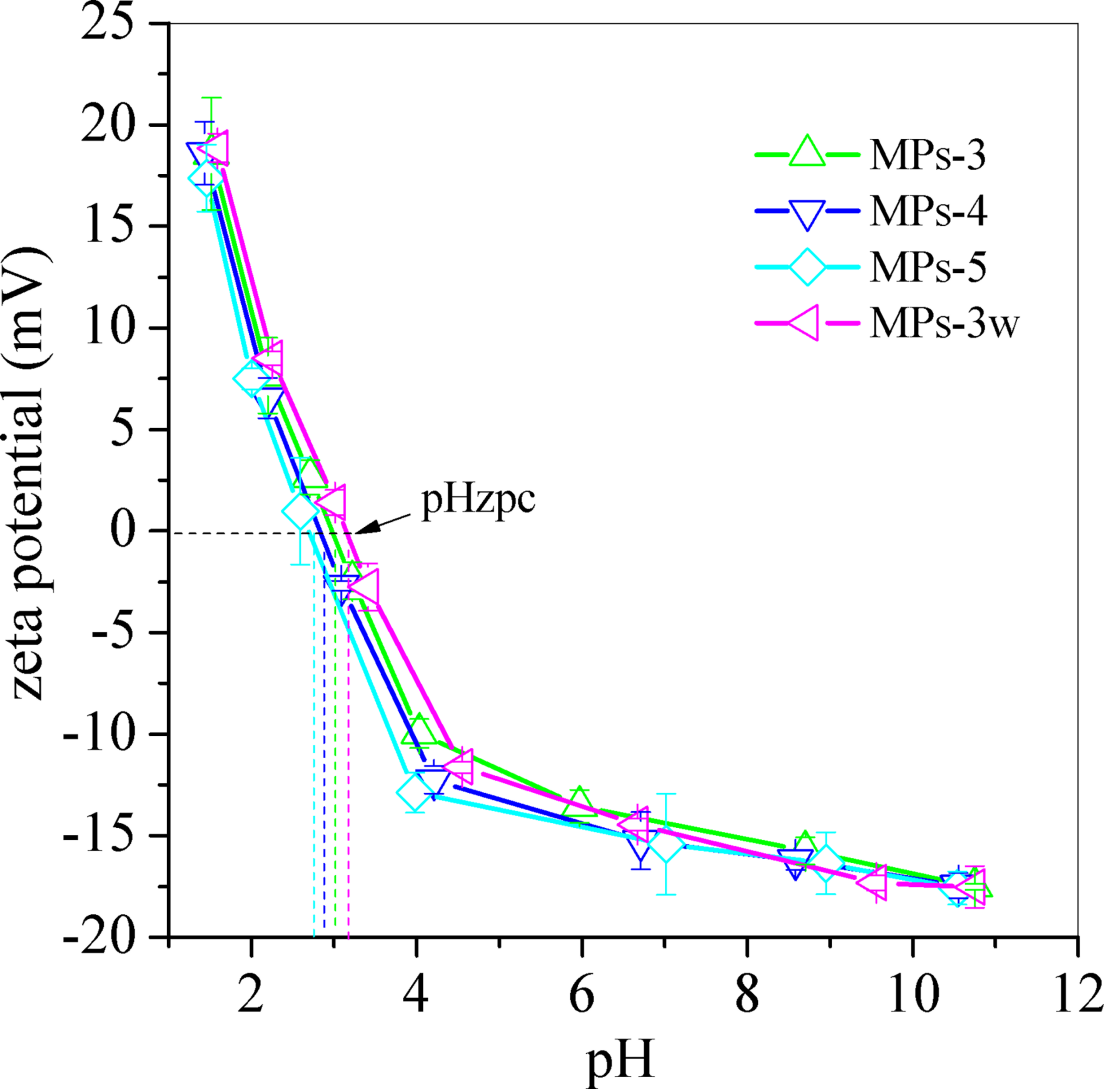
zeta potential variation of MPs. Herein, MPs 3–5 were synthesized by changing the molar ratio of ascorbic acid to Fe^3+^ from 0.1 to 0.15, 0.2, separately. MPs-3w was prepared at the molar ratio of 0.1 by acid wastewater digestion.

The zeta potentials of MPs were shown in Fig 11 and pHzpc of MPs was calculated at the zeta potential of 0 mV. The pHzpc for MPs-3w and MPs 3–5 was 3.15, 2.98, 2.83 and 2.69, respectively. During the batch adsorption of MB, the pH of MB solution changed between 7.1 and 7.3, and thus all MPs have negative charges on their surfaces. The pK_1_ of MB was 4.5 [44], At pH > pK_1_, the = N–in MB molecules was protonated and the cationic MB was formed. Therefore, the cationic MB was adsorbed onto the negative charged surface of MPs by electrostatic attraction.

**Fig 12:**
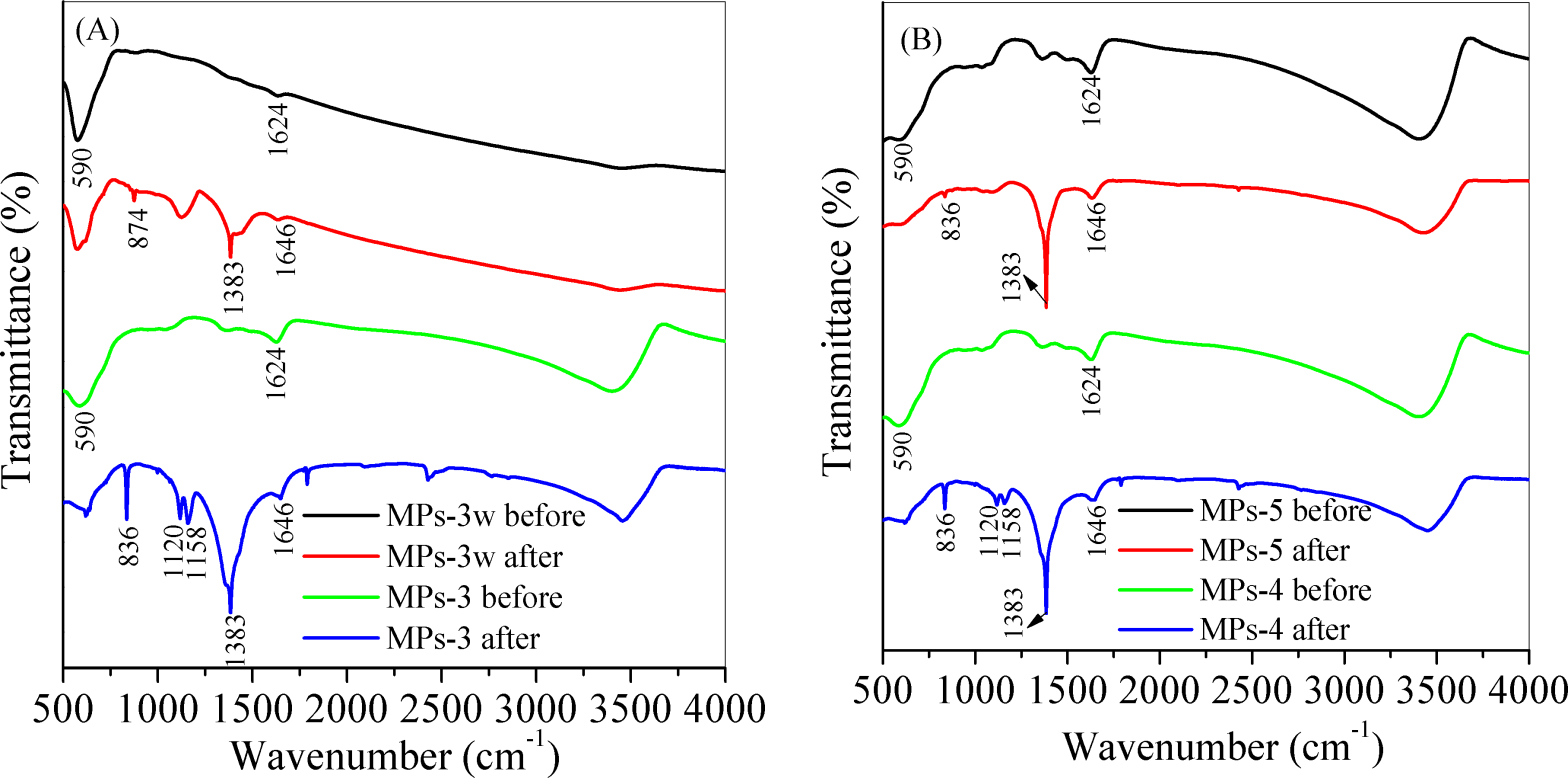
**Infrared spectra of MPs-3w and MPs-3w (A), and MPs-4 and MPs-5 (B) before and after MB adsorption.** Herein, MPs 3–5 was synthesized by changing the molar ratio of ascorbic acid to Fe^3+^ from 0.1 to 0.15, 0.2, respectively. MPs-3w was prepared at the molar ratio of 0.1 by acid wastewater digestion.

To determine the interaction of MB with MPs during adsorption, the infrared spectra of MB adsorbed onto MPs-3 and MPs-3w was analyzed as shown in Fig 12A. It was demonstrated in the MPs-3 and MPs-3w spectrum that the peaks at 590 cm^-1^ corresponded to the stretching mode of Fe-O in Fe_3_O_4_, and peaks at 1624 cm^-1^ were related to the O-H functional groups [45]. After MB was adsorbed, the peaks of O-H groups shifted from 1624 cm^-1^ to 1646 cm^-1^, which indicated the O-H group participated in MB adsorption. The peaks appeared at 836, 874 and 1383 cm^-1^ were ascribed to wagging vibration of C-H in the alkyl group and aromatic ring of MB, respectively [46]. Other peaks appeared at 1159 cm^-1^ and 1118 cm^-1^ after adsorption were attributed to the asymmetric vibration of C = S and stretching vibrations of C-S-C, respectively [46]. This indicated that MB was adsorbed onto MPs. The infrared spectra of MB adsorbed onto MPs-4 and MPs-5 in Fig 12B showed similar peaks with MPs-3.

Three typical functional groups of ≡FeOH_2_^+^, ≡FeOH and ≡FeO^-^ were generated and present on the surface of MPs [47]. These functional groups were mostly ionized to the negatively charged ≡Fe-O^-^ at alkaline condition and showed weakly acidic ion exchange property. Under alkaline conditions, it was attached by Na^+^ to form ≡FeO-Na^+^ through electrostatic forces [38, 48]. Na^+^ had relative lower affinity to the ≡Fe-O^-^ functional group and the attached Na^+^ could be favorably replaced by MB [37, 47]. The possible reactions related to the MB adsorption are described below:

$$\equiv FeOH+NaOH\to \equiv FeONa+H_{2}O$$ (26)

$$\equiv FeONa+MB^{+}\to \equiv FeOMB+Na^{+}$$ (27)

## Conclusion

Iron mud, a negatively valued waste from groundwater treatment plant, was successfully converted to a magnetic cation exchanger for MB adsorption by simple Fe^3+^/Fe^2+^ coprecipitation with H_2_A as reduction reagent. The synthesized MPs-3 with the H_2_A/Fe^3+^ ratio of 0.1 had an Ms of 4.6 emu/g and highest MB adsorption capacity of 87.3 mg/g. The adsorption of MB onto MPs was in agreement with the Langmuir isotherm model and the adsorption kinetics fitted well with the pseudo-second-order model. When the dissolving reagent of nitric acid was replaced by acid wastewater, the generated MPs-3w also showed significant magnetic response and MB adsorption capacity of 56.7 mg/g. Our study demonstrated that cation exchange and electrostatic attraction, not surface area, were the major mechanism for MB adsorption. The magnetic adsorbent synthesized from negatively-valued iron mud waste using an environment-friendly method had a good potential for effective treatment of dye wastewater.

## Supporting information

S1 Table Characteristics of the acid wastewater and the supernatant. (Unit: Mg/L).
(DOC)

S2 Table Parameters and the regression coefficients (R^2^) of the isotherm models. (DOC)

S3 Table The cost for MPs-3 synthesis. (DOC)

## Abbreviations

H_2_A: ascorbic acid
HA^-^: ascorbate
2,3-DKG: _L_-diketogulonate
DHA: dehydroascorbic acid
GAC: *HA* granule active carbon
Fe(ACAC)_3_: iron(III) acetylacetonate
MB: methylene blue
MPs: magnetic particles
Ms: saturation magnetization
FWHM: full-width-at-half-maximum
FE-SEM: field emission scanning electron microscope
VSM: vibrating sample magnetometer
XPS: X-ray photoelectron spectroscopy
XRD: X-ray diffraction
BET: Brunauer-Emmett-Teller
CEC: cation exchange capacity
