# Supplementary material for: Synthesis and characterization of a magnetic adsorbent from negatively-valued iron mud for methylene blue adsorption
Source: PLoS One. 2018 Feb 2;13(2):e0191229. doi: 10.1371/journal.pone.0191229 (PMC5796699; doi:10.1371/journal.pone.0191229)
Supplement: S1 Table — (Unit: Mg/L). (DOC) [file pone.0191229.s001.doc]

**S1 Table. Characteristics of the acid wastewater and the supernatant. (Unit**: mg/L).

|  | **pH** | **Fe** | **Al** | **Na^+^** | **NH_4_^+^** | **K^+^** | **Ca^2+^** | **Mg^2+^** | **Cl^-^** | **NO_3_^-^** | **SO_4_^2-^** | **TOC** |
| --- | --- | --- | --- | --- | --- | --- | --- | --- | --- | --- | --- | --- |
| **Acid wastewater** | 0.61 | 185.9 | 132.8 | 1350.6 | 194.6 | 304.3 | 3736.1 | 507.7 | 3992.5 | 444.4 | 22492.2 | 453.05 |
| **Supernatant of iron mud mixed with acid wastewater** | 1.21 | 3962.8 | 12.2 | 1582.7 | 232.4 | 72.7 | 993 | 560.8 | 3987.4 | 435.8 | 16438.6 | 903.75 |
| **Supernatant after the coprecipitation process** | 9.5 | 19.4 | 0 | - | 203.5 | 72.6 | 287.5 | 160.9 | 3749.4 | 412.1 | 12953.5 | 677 |
