# Supplementary material for: Synthesis and characterization of a magnetic adsorbent from negatively-valued iron mud for methylene blue adsorption
Source: PLoS One. 2018 Feb 2;13(2):e0191229. doi: 10.1371/journal.pone.0191229 (PMC5796699; doi:10.1371/journal.pone.0191229)
Supplement: S2 Table — (DOC) [file pone.0191229.s002.doc]

**S2 Table. Parameters and the regression coefficients (R^2^) of the isotherm models**.

| **Isotherm** | **Parameters** | **MPs-3** | **MPs-4** | **MPs-5** | **MPs-3w** | **GAC** |
| --- | --- | --- | --- | --- | --- | --- |
| **Langmuir** | R^2^ | 0.997 | 0.997 | 0.998 | 0.998 | 0.998 |
|  | *q_m_* (mg/g) | 87.34 | 61.84 | 26.97 | 56.69 | 20.53 |
|  | *K_L_* | 4.734 | 3.498 | 1.37 | 2.707 | 1.114 |
|  | *a_L_* | 0.054 | 0.057 | 0.051 | 0.048 | 0.054 |
| **Freundlich** | R^2^ | 0.907 | 0.957 | 0.891 | 0.942 | 0.835 |
|  | *b_F_* | 0.43 | 0.36 | 0.38 | 0.39 | 0.34 |
|  | *K_F_* | 9.63 | 9.45 | 3.55 | 7.26 | 3.29 |
| **Redlich-Peterson** | R^2^ | 0.996 | 0.999 | 0.995 | 0.999 | 0.997 |
|  | *K_R_* | 5.56 | 4.25 | 1.7 | 3.21 | 1.42 |
|  | *α_R_* | 0.084 | 0.091 | 0.084 | 0.075 | 0.092 |
|  | *β _R_* | 0.95 | 0.95 | 0.95 | 0.95 | 0.95 |
| **Templin** | R^2^ | 0.944 | 0.983 | 0.95 | 0.984 | 0.94 |
|  | B | 16.01 | 10.42 | 5.33 | 10.24 | 3.81 |
|  | A | 0.858 | 1.195 | 0.583 | 0.785 | 0.795 |
| **Sips** | R^2^ | 0.984 | 0.992 | 0.996 | 0.992 | 0.988 |
|  | 1/n | 0.713 | 0.706 | 0.935 | 0.845 | 0.981 |
|  | *q_m_* (mg/g) | 113.1 | 64.8 | 28.9 | 55.1 | 22.1 |
|  | *b_S_* | 0.022 | 0.05 | 0.038 | 0.053 | 0.042 |
| **Toth** | R_2_ | 0.978 | 0.996 | 0.981 | 0.993 | 0.986 |
|  | t | 0.361 | 0.258 | 0.332 | 0.287 | 0.392 |
|  | *K_T_* | 156.3 | 159.8 | 57.2 | 142.7 | 32.9 |
|  | *a_T_* | 1.99 | 1.16 | 1.8 | 1.51 | 1.89 |
