# Supplementary material for: Synthesis and characterization of a magnetic adsorbent from negatively-valued iron mud for methylene blue adsorption
Source: PLoS One. 2018 Feb 2;13(2):e0191229. doi: 10.1371/journal.pone.0191229 (PMC5796699; doi:10.1371/journal.pone.0191229)
Supplement: S3 Table — (DOC) [file pone.0191229.s003.doc]

**S3 Table. The cost for MPs-3 synthesis.**

| **Reagent and Energy** | **Prize per ton**  **(US$)** | **Usage**  **per 0.8 kg iron mud** | **Prize**  **per 0.8 kg iron mud**  **(US$)** | **Total prize**  **per ton MPs-3**  **(US$)** |
| --- | --- | --- | --- | --- |
| **nitric acid** | 77.27 | 1000 mL | 0.08 | 148.6 |
| **ascorbic acid** | 909.09 | 41.8 g | 0.04 | 73.08 |
| **sodium hydroxide** | 227.27 | 350 g | 0.08 | 152.97 |
| **power** | 0.23 US$/Kw.h | 0.8 Kw.h | 0.15 | 279.72 |
| **MPs-3 per ton** |  |  |  | 654.37 |
